# How well do genetic markers inform about responses to intraspecific admixture? A comparative analysis of microsatellites and RADseq

**DOI:** 10.1186/s12863-021-00974-3

**Published:** 2021-06-28

**Authors:** Yeşerin Yıldırım, Anders Forsman, Johanna Sunde

**Affiliations:** Centre for Ecology and Evolution in Microbial Model Systems, EEMiS, Department of Biology and Environmental Science, Linnaeus University, SE-392 31 Kalmar, Sweden

**Keywords:** *Esox lucius*, Hybridization, Interbreeding, Microsatellites, Offspring performance, Outbreeding, Pike, RADseq, SNP

## Abstract

**Background:**

Fitness consequences of intraspecific genetic admixture can vary from positive to negative depending on the genetic composition of the populations and environmental conditions. Because admixture has potential to influence the success of management and conservation efforts, genetic similarity has been suggested to be used as a proxy to predict the outcome. Studies utilizing microsatellites (a neutral marker) to investigate associations between genetic distance and admixture effects show conflicting results. Marker types that yield information on genome-wide and/or adaptive variation might be more useful for predicting responses to inter-population hybridization. In this study we utilized published data for three populations of pike (*Esox lucius*) to investigate associations between offspring performance (hatching success) and parental genetic similarity in experimentally purebred and admixed families, based on neutral (microsatellites), genome-wide neutral (RADseq SNPs), and adaptive (SNPs under selection) markers.

**Results:**

Estimated similarity varied among the markers, likely reflecting differences in their inherent properties, but was consistently higher in purebred than admixed families. A significant interaction between marker type and admixture treatment reflected that neutral SNPs yielded higher estimates than adaptive SNPs for admixed families whereas no difference was found for purebred families, which indicates that neutral similarity was not reflective of adaptive similarity. When all samples were pooled, no association between similarity and performance was found for any marker. For microsatellites, similarity was positively correlated with hatching success in purebred families, whereas no association was found in admixed families; however, the direction of the effect differed between the population combinations.

**Conclusions:**

The results strengthen the notion that, as of today, there is no proxy that can reliably predicted the outcome of admixture. This emphasizes the need of further studies to advance knowledge that can shed light on how to safeguard against negative consequences of admixture, and thereby inform management and promote conservation of biological diversity.

**Supplementary Information:**

The online version contains supplementary material available at 10.1186/s12863-021-00974-3.

## Background

Intraspecific genetic admixture (henceforth ‘admixture’) occurs when separated populations starts interbreeding [1]. It occurs naturally in many terrestrial and aquatic species in the wild as a consequence of dispersal [2], and can also occur as a result of anthropogenic activities, for example management actions to support populations (e.g. supplementations and translocations) [3, 4], removal of dispersal barriers [5], and escapes of farmed individuals [6].

Admixture affects the genetic composition of the involved populations and tends to increase the genetic variation in the receiving population. Increased genetic variation is generally considered to positively influence aspects of population performance. For example, it has been shown that genetically and phenotypically more diverse populations are better able to cope with environmental change, and to colonize novel environments [2, 7–10]. Admixture can also have positive fitness effects by allowing creation of novel gene combinations and dampening inbreeding depression by masking detrimental effects of deleterious recessive alleles (heterosis) [1, 3]. However, the influx of new genetic material can also result in negative fitness effects. If the involved populations are highly differentiated, genomic incompatibilities might exist (e.g. as a result of chromosomal rearrangements in the parental populations) [11, 12], and admixture may thus lead to outbreeding depression. In addition, admixture between populations that have adapted to different environmental conditions might dilute favorable alleles [1], give rise to offspring with intermediate phenotypes that are not optimal in either of the parental environments [4, 13], and has potential to impair fitness in subsequent generations by breaking up co-adapted gene complexes or by underdominance (i.e. heterozygote disadvantage) [1, 4, 13–15]. The net outcome (with regards to both magnitude and direction) of the response to admixture will be determined by an interplay between these mechanisms, and will thus depend on the genetic composition of the parental populations, local adaptations and environmental conditions [13].

The motivation for management and conservation efforts resulting in admixture is to increase productivity, viability, and adaptability of populations [3, 11, 14]. To avoid undesirable outcomes of such efforts, it would be valuable to have a reliable proxy that could be used to predict the response to admixture. Genetic similarity has been put forward as such a candidate proxy [11, 14]. However, empirical studies show conflicting results [3, 11, 16–19], and further studies are therefore required to increase the understanding about potential associations between genetic similarity and the response to admixture, and to understand the reasons for the observed inconsistencies. Previous studies have commonly estimated genetic similarity based on a modest number (4–32) of microsatellite markers [20–23]. Microsatellites are mainly neutral markers that only occasionally reflect functional evolution, e.g. by residing within coding or regulatory regions or by being linked to functional loci [24, 25]. Microsatellites have high mutation rates, and loci with relatively high allelic variation are commonly selected during the marker development [26, 27]. As a consequence, genetic diversity estimates based on small numbers of microsatellites generally do not reflect the genome-wide diversity [26, 28]. It is therefore possible that the inconsistencies in associations between genetic similarity and admixture effects in previous studies [3, 11, 14, 16, 20, 29] can be partly attributed to the use of non-representative estimates of genetic diversity, and estimates of genome-wide diversity might be better able to predict the outcome of admixture. In addition, estimates of parental genetic similarity might be more informative than population-based similarity, as the former accounts for the inter-individual variation [23].

The development of next generation sequencing techniques, such as Restriction-site associated DNA sequencing (RADseq) has enabled to genotype many markers at a low cost, even for non-model species [30]. RADseq commonly yields thousands of single nucleotide polymorphisms (SNPs), which provides better estimates of genome-wide diversity. It has also been shown that even relatively low numbers of SNPs (≥50) generally has the same, if not more, statistical power compared to 20 microsatellites in relatedness studies [31], and that RADseq SNPs generally tend to outperform microsatellites in population genetic studies [32], but see [33]. In addition, it is functional – not neutral - genetic variation that is key for the adaptive potential of populations [34] with potential to influence the outcome of admixture. It can therefore be hypothesized that estimates of genetic similarity based on adaptive genetic variation (or divergence) offer a better predictor of the response to admixture [35]. RADseq SNPs thus offer a viable alternative as they can provide information on both neutral and adaptive genetic variation and differentiation.

The overall aim of the present study was to evaluate the potential of neutral (microsatellites), genome-wide neutral (RADseq SNPs), and adaptive (outlier SNPs under selection) parental genetic similarity to predict admixture effects on offspring performance (hatching success) resulting from experimental matings of individuals representing genetically separated and locally adapted natural populations. To this end, we used previously published genetic and phenotypic data for three populations (Harfjärden, Lerviksbäcken, and Oknebäck; henceforth Harfjärden, Lervik and Okne) of anadromous Baltic Sea pike (*Esox lucius*) [32, 36]. Pike is a long-lived fish, which is both ecologically and socio-economically important. Unfortunately, it has suffered decreases during the last decades, and has therefore been the target of extensive management efforts [37–40]. All three populations included in this study are neutrally genetically differentiated based on both microsatellites [32, 39, 41] and RADseq SNPs [32], but the degree of differentiation differ among the population pairs. Differentiation between the two adjacent streams (Lervik and Okne) is low (*F*_*ST*_ = 0.044–0.071), and some gene flow may occur [32, 39, 41]. Comparisons of the geographically more separated populations (Harfjärden compared to both other populations), showed no signs of gene flow and higher differentiation (*F*_*ST*_ = 0.136–0.226) [32, 39, 41]. Studies also indicate that the populations are adaptively differentiated and display different local adaptations [36, 42–45]. More specifically, we investigated whether: *i*) variation in parental similarity estimates among pairs of experimentally mated males and females were consistent or differed among the markers; *ii*) parental similarity was associated with offspring hatching success; and *iii*) the associations between parental similarity and offspring performance were consistent or depended on whether parental similarity was estimated based on neutral (microsatellites), genome-wide neutral (RADseq SNPs) or adaptive (RADseq SNPs under selection) genetic markers.

## Results

### Comparison of parental similarity based on microsatellites and RADseq SNP data

**Fig. 1:**
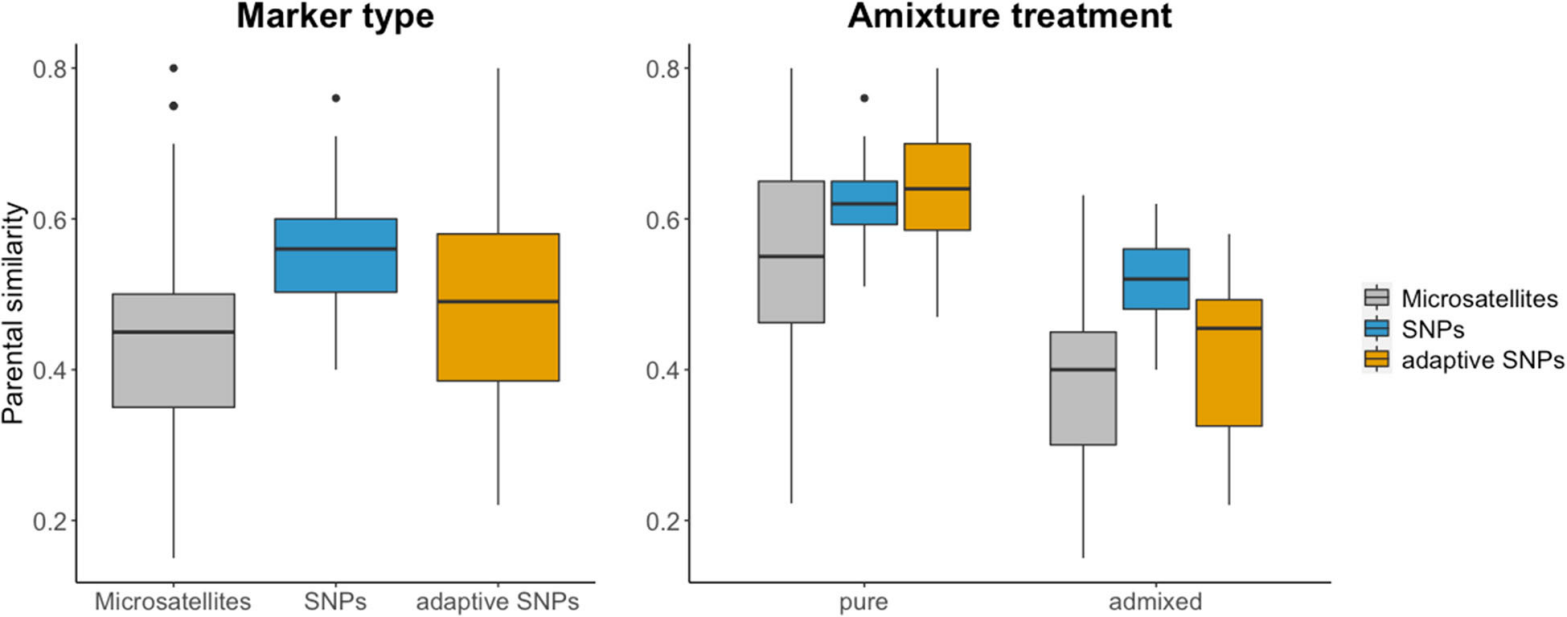
Estimates of pairwise parental similarity (proportion of alleles shared between individuals) for the same set of individuals (*N* = 64). Estimates are based on three different datasets: neutral (microsatellites), genome-wide neutral (RADseq SNPs), and adaptive (outlier RADseq SNPs under selection). Left panel show estimates for all families, and the right panel show estimates for the two admixture treatments (purebred and admixed) separated

Estimates of pairwise parental similarity based on the three different datasets (microsatellites, neutral SNPs and adaptive SNPs) differed (*F*_*2,178*_ = 36.38, *P* < 0.001, Fig. 1), and differences were evident between all pairwise comparisons (Tukey’s test: microsatellites – adaptive SNPs: *P* = 0.014; microsatellites – neutral SNPs: *P* < 0.001; adaptive SNPs – neutral SNPs: *P* = 0.003). Estimated parental similarity was highest for neutral SNPs (mean ± SD: 0.55 ± 0.07), intermediate for adaptive SNPs (mean ± SD: 0.49 ± 0.15), and lowest for microsatellites (mean ± SD: 0.43 ± 0.15). In addition, the range of similarity estimates was smaller for neutral SNPs than for both microsatellites and adaptive SNPs (Fig. 1).

Parental similarity estimates were higher for purebred families than for admixed families for all three datasets (Student’s t-test: microsatellites: *t* = − 5.88, df = 49.39, *P* < 0.001; neutral SNPs: *t* = − 9.63, df = 66.36, *P* < 0.001; adaptive SNPs: *t* = − 11.61, df = 72.61, *P* < 0.001) (Fig. 1). In addition, the analysis revealed a significant interaction effect between marker type and admixture treatment (purebred or admixed) (*F*_2,176_ = 10.16, *P* < 0.001), which reflected that neutral SNPs yielded higher estimates for the admixed families than did microsatellites and adaptive SNPs (Tukey’s test: microsatellites – adaptive SNPs: *P* = 0.15; microsatellites – neutral SNPs: *P* < 0.001; adaptive SNPs – neutral SNPs: *P* < 0.001), whilst there was no difference between neutral SNPs and adaptive SNPs for purebred families (Tukey’s test: microsatellites – adaptive SNPs: *P* < 0.001; microsatellites – neutral SNPs: *P* = 0.008; adaptive SNPs – neutral SNPs: *P* = 0.70) (Fig. 1).

### No association between parental similarity and hatching success

**Fig. 2:**
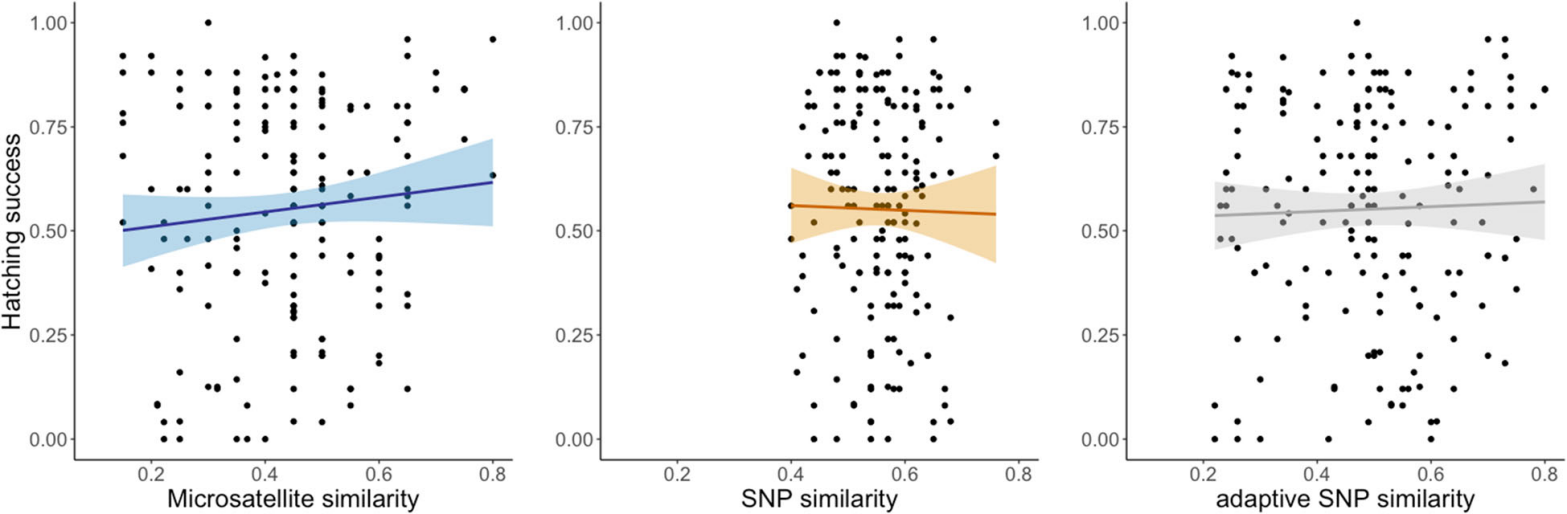
Relationship between hatching success and pairwise parental similarity estimated based on the three different datasets: microsatellites (left plot), RADseq SNPs (middle plot), and adaptive (outlier) RADseq SNPs (right plot)

When all the samples were pooled, a large variation in hatching success was evident throughout the range of parental similarity for all three datasets (Fig. 2). Parental similarity was not associated with hatching success for any of the marker types (microsatellites: *F*_1,177_ = 1.23, *P* = 0.27, neutral SNPs: *F*_1,177_ = 0.04, *P* = 0.83, adaptive SNPs: *F*_1,177_ = 0.27, *P* = 0.61).

**Fig. 3:**
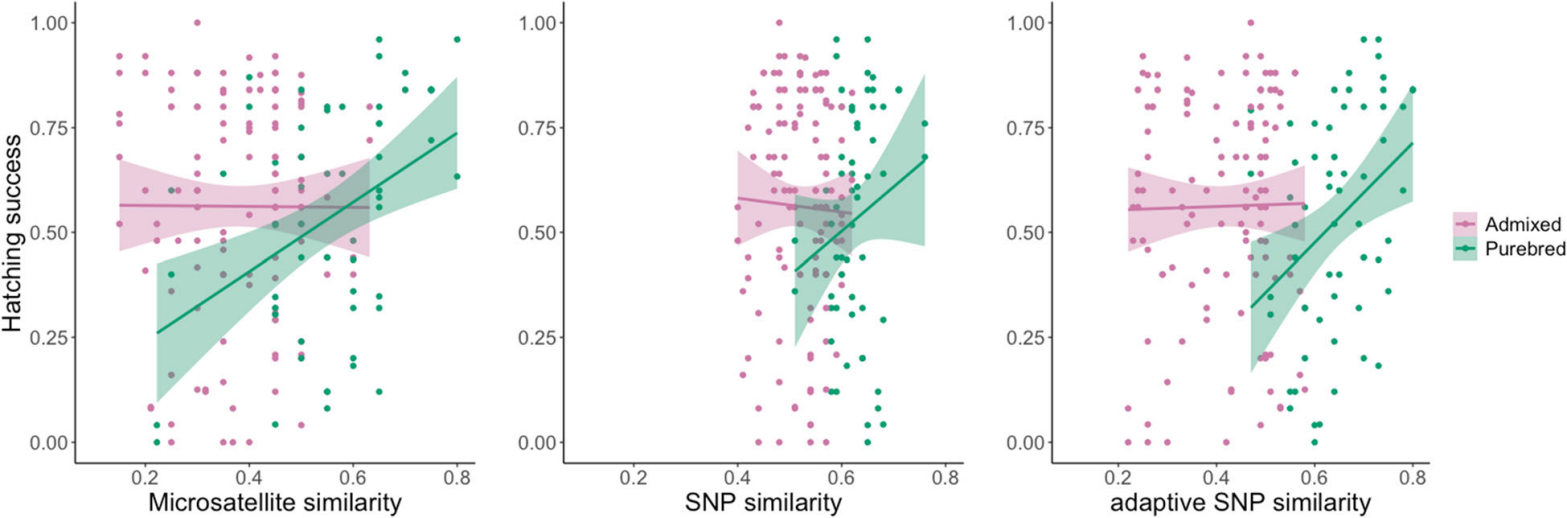
Relationship of purebred (green) and admixed (purple) families between hatching success and pairwise parental similarity estimated based on three different datasets: microsatellites (left plot), RADseq SNPs (middle plot), and adaptive (outlier) RADseq SNPs (right plot)

When the samples were classified according to admixture treatment (purebred or admixed), to test whether associations between parental similarity and hatching success differed between purebred and admixed families, no significant interaction effect was found for either neutral SNPs (effect of treatment: *F*_1,176_ = 0.24, *P* = 0.62; effect of parental similarity: *F*_1,176_ = 0.045, *P* = 0.85; effect of interaction between parental similarity and admixture treatment: *F*_1,175_ = 0.67, *P* = 0.41) or adaptive SNPs (effect of treatment: *F*_1,176_ = 1.83, *P* = 0.18; effect of parental similarity: *F*_1,176_ = 0.27, *P* = 0.18; interaction between parental similarity and admixture treatment: *F*_1,175_ = 3.07, *P* = 0.08) (Fig. 3). However, for the microsatellite data, there was a significant effect of the interaction between admixture treatment and parental similarity (*F*_1,175_ = 4.31, *P* = 0.04), reflecting that there was a positive relationship between similarity and hatching success for purebred families (a positive slope), whereas parental similarity was not associated with hatching success in the admixed families (Fig. 3). Although not statistically significant, similar trends (a positive slope for purebred families and no association for admixed families) were observable also for the two other marker types (neutral SNPs and adaptive SNPs) (Fig. 3).

**Fig. 4:**
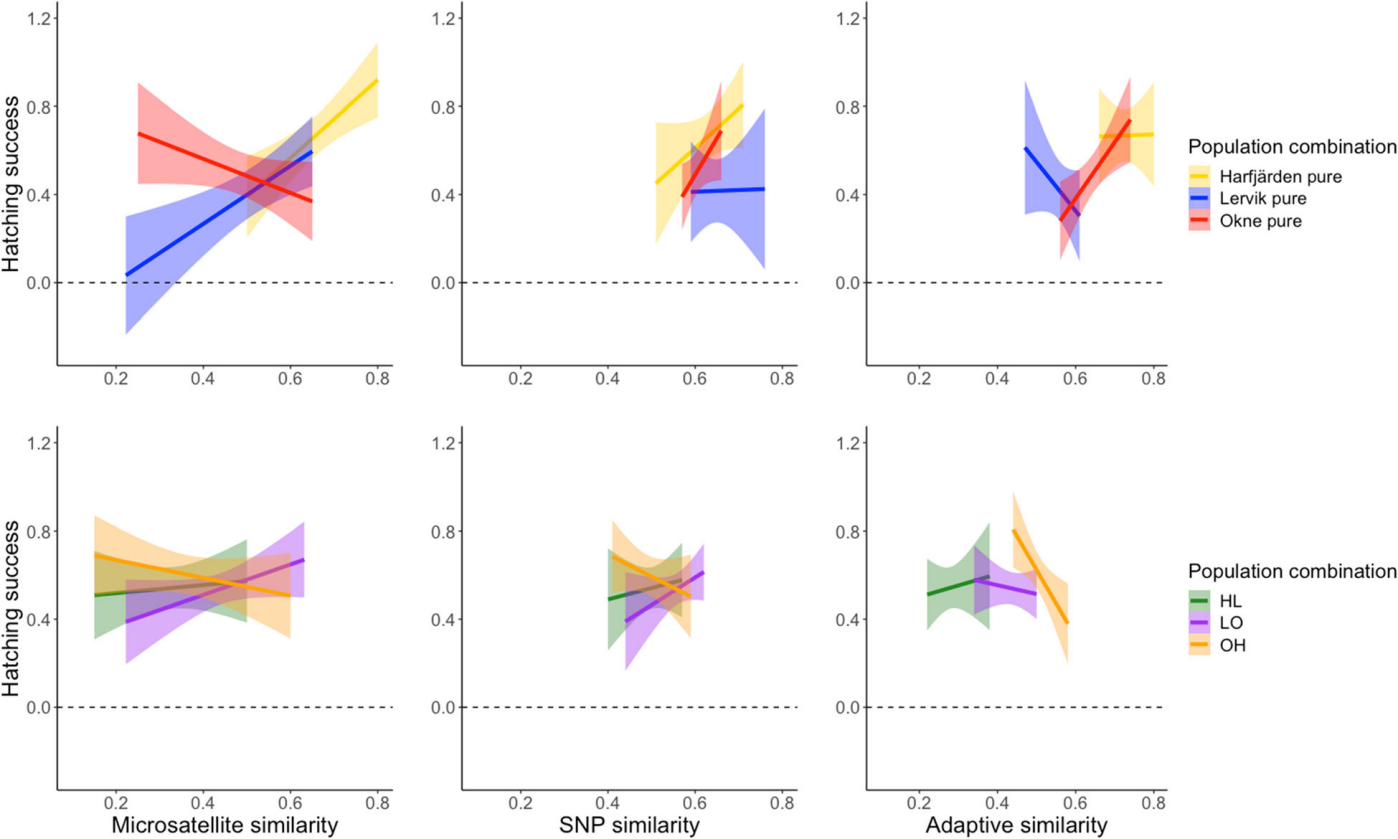
Relationship between hatching success and pairwise parental similarity estimated based on microsatellites (left column), RADseq SNPs (middle column), and adaptive (outlier) RADseq SNPs (right column) for purebred (top row) and admixed (bottom row) population combinations: based on three different datasets

When the data was further separated into specific population combinations (3 purebred and 3 admixed groups, based on the source population of each of the parental individuals) (Fig. 4), Student’s T-tests showed that the association between parental similarity and hatching success differed among the purebred populations for the microsatellite dataset (*F*_1,2_ = 7.47, *P* < 0.001), but not for the neutral SNP dataset (*F*_1,2_ = 0.72, *P* = 0.49) whereas adaptive SNPs approached marginal significance (*F*_1,2_ = 2.81, *P* = 0.061). No significant interaction effects between parental similarity and population combination were found for the admixed population combinations for any of the marker types (microsatellites: *F*_1,2_ = 0.95, *P* = 0.3880; neutral SNPs: *F*_1,2_ = 0.81, *P* = 0.446; adaptive SNPs: *F*_1,2_ = 1.73, *P* = 0.177).

## Discussion

Increased knowledge about the effects of admixture can further the understanding about evolution of genetic structure and what shapes patterns of diversity, and also help avoid undesirable effects associated with conservation measures and management actions. In the present study, we used data for pike to evaluate whether estimates of parental genetic similarity based on three different marker types (microsatellites, neutral SNPs and adaptive SNPs) could predict the outcome of admixture. To our knowledge, this is the first attempt to systematically evaluate and compare the utility of different markers as proxies of parental compatibility and predictors of offspring performance. The main findings were that: *i*) estimated parental similarity differed between the marker types, and were consistently higher for purebred families compared to admixed families; *ii*) parental similarity was not consistently or clearly associated with hatching success for any of the marker types; and *iii*) the association between hatching success and parental similarity as estimated based on microsatellites was different for purebred and admixed groups, and also differed between population combinations.

### Parental similarity differed between marker types

The findings that both values and ranges of estimated parental similarity differed between the marker types (Fig. 1) were expected, and likely reflect differences in the inherent properties of the marker types and datasets. The higher similarity estimates obtained for both of the SNP datasets (genome-wide neutral and adaptive) compared to microsatellites (neutral) is likely explained by the higher number of alleles per locus for microsatellites [26, 27], which might lead to overestimation of differentiation between individuals [26]. Moreover, the denser RADseq SNP data (~ 1500 SNPs) is more likely to represent genome-wide diversity than the moderate number of microsatellites [28]. The wider range of similarity for both microsatellites and adaptive SNPs compared to neutral SNPs likely in part reflect the relatively low number of loci used for the former two, as each allele will have a large effect on the estimated similarity. In addition, the adaptive dataset consists of loci that are associated with environmental variables (temperature and salinity). It is therefore likely that the wide range for adaptive SNPs also in part reflects the range of environmental differences among the populations. Taken together, this calls for caution when comparing results of studies that have used different markers, and comparisons should be based on ranking rather than absolute values.

The finding that purebred families had higher parental similarity than admixed families for all datasets (Fig. 1) was also expected as the three study populations are both neutrally and adaptively differentiated [32, 41, 46]. That similarity estimates were higher for neutral SNPs than for adaptive SNPs for admixed families but did not differ for purebred families, likely reflects differences in neutral and adaptive evolution. Neutral loci are mainly affected by neutral and stochastic processes, whereas functional loci are also affected by deterministic processes such as selection [30, 47]. For admixed families, the loci in the adaptive dataset are probably under diversifying selection (as the algorithm used in the study identifies the outlier loci associated with environmental variables [48]), which would explain the higher degree of differentiation observed for adaptive SNPs.

### No association between parental similarity and hatching success

While there was no overall association between hatching success and parental similarity for any of the marker types (Fig. 2), the results indicated that the effect of parental similarity might differ between purebred and admixed families, and that the association was positive only for purebred families (Fig. 3). The large variation in hatching success for both admixed and purebred families throughout the similarity ranges for all marker types (Fig. 3) and the lack of any consistent association across marker types between parental similarity and offspring hatching success argues against the utility of parental genetic similarity estimates as a reliable predictive proxy for admixture effects in this system. It is possible that the range of genetic differentiation between the study populations was not wide enough to get a complete picture, that effects of admixture manifest more strongly in natural environments likely reflecting differences in the selective regimes [49–51], and that some admixture effects are not expressed until the F2 generation [10, 14, 16]. That our analyses, like some previous investigations of other species [17–20, 22, 23, 52], failed to detect any association between parental similarity and offspring performance cannot be taken as evidence that the genetic resemblance between parents is of no importance. However, that such an association apparently is difficult to detect is both disappointing and problematic, particularly from an applied conservation perspective.

Sadly, the conclusion that parental similarity is a poor predictor of the response to admixture extends to other candidate proxies, such as geographic distance, neutral genetic differentiation, genetic diversity or environmental similarity. Empirical studies using these different proxies [3, 11, 14, 16–18, 29, 53, 54] show conflicting results. This inconsistency is likely reflective of the complex interactions between environmental factors and inherent properties of the parental populations [15, 54].

## Conclusions

The present study showed that parental similarity was not consistently or clearly associated with hatching success for any of the marker types (neutral, genome-wide neutral, and adaptive). Our present study thus strengthens the conclusion that, as of today, there is no proxy that can reliably predict the outcome of admixture. There is therefore a clear need for further studies and different approaches to advance knowledge that can shed light on how to safeguard against negative consequences of admixture, and thereby inform management and promote successful conservation of biological diversity.

## Methods

### Study species

Pike is a long-lived fish that inhabits both freshwater and brackish water systems [55]. As one of the most common large predatory fishes in the Baltic Sea, it fills an important function in many systems where it regulates the abundance of species in lower trophic levels through top-down trophic cascades [56, 57]. As a valued species in both commercial and recreational fishing, pike is also socio-economically important [37, 58]. It has also emerged as a model species for studies of ecology and evolution [59]. Unfortunately, pike populations in the Baltic Sea have been experiencing declines during the last decades [37, 38, 57, 60]. Several different factors, such as eutrophication, habitat loss, overfishing, and altered species interactions, have been proposed to have contributed to the decrease [37, 38, 57, 61, 62]. Due to the importance of pike, management actions, e.g., restoration of spawning locations (wetlands), and large-scale stocking programs have therefore been carried out to support and revitalize the populations [39, 40, 63, 64].

### Study populations

The three populations of anadromous pike included in this study reproduce in different spawning habitats in the Kalmar Sound region [36]. Two of the localities (Lervik and Okne) are closely located (approximately 20 km shortest waterway distance) in the southeast of the Swedish mainland (Lervik: N57° 04.414′; E16°31.246′, Okne: N57° 01.200′; E16° 26.700′), and the third locality (Harfjärden) is located on the east coast of the island of Öland (N56° 49.063′; E16° 48.673′; approximately 120 and 135 km from Lervik and Okne, respectively) [32]. All three populations are significantly genetically differentiated from each other as indicated by results from analyses based on both microsatellites [32, 39, 41] and RADseq SNPs [32]. Among the three populations, Harfjärden forms the most distinct genetic cluster with high genetic differentiation compared to both other populations (*F*_*ST*_ = 0.136–0.226, *P* < 0.01) [32, 39, 41] and no evidence of gene flow to the Swedish mainland populations [32]. Despite evidence of low levels of gene flow, the two closely located streams (Lervik and Okne) also form genetically distinct populations but with low differentiation (*F*_*ST*_ = 0.044–0.071, *P* < 0.01) [32, 39, 41], and the distinctiveness of the clusters becomes more evident with increasing numbers of samples/loci included in the analyses [32, 41]. In addition, common garden and translocation experiments indicate that the study populations exhibit local adaptations for several traits including early life history traits and reproductive investment [42], salinity [36] and temperature tolerance [45], growth rate and adult body size [43] and vertebrae count [44]. The local adaptations have been attributed to environmental differences among the three spawning grounds, such as differences in the salinity and temperature regimes [45, 65], and in the amount of suspended materials [42]. Moreover, the study populations (exactly the same set of individuals as in the present study) exhibited genetic signatures of selection associated with salinity and temperature [32].

### Estimates of hatching success

We obtained estimates of hatching success from the study by Sunde, et al. [36] that investigated effects of admixture on F1 offspring performance [46]. More specifically, the study investigated whether and how admixture affected offspring quality in different population combinations, and whether the effects were population-specific [36]. In that study, gametes were collected from a total of 66 individuals from the three populations (Lervik, Okne and Harfjärden). To include the exact same set of individuals in both the hatching success dataset and genotyping dataset in the present study, we decided to omit two of the samples (that did not pass the quality control in the RADseq pipeline) before proceeding with the analyses (see the subsection Estimates of parental similarity below), thus resulting in a total of 64 parental individuals (Harfjärden: *N*_males_ = 12, *N*_females_ = 10, Lervik: *N*_males_ = 12, *N*_females_ = 9, Okne: *N*_males_ = 10, *N*_females_ = 11). In short, separate batches of eggs from each female were artificially fertilized with milt from one male from each population, thus creating one purebred (male from the same population) and two admixed (male from one of the other populations) treatments per female. Each combination was done in duplicates, and this resulted in a total of 180 units from 90 female/male pairs (30 purebred and 60 admixed). Eggs, and subsequently hatched fry were reared in a common garden environment, and three offspring performance measures: 1) hatching success (proportion of eggs that hatched), 2) fry survival (15 days following hatching), and 3) fry body length (at 15 days post hatch) were estimated (for details see [36]). Because Sunde, et al. [36] found that hatching success was affected by the treatment we chose not to include the other offspring performance measures in the present study, to avoid using potentially biased estimates and low statistical power resulting from differences in hatching success, and the associated unequal and small sample sizes.

### Estimates of parental similarity

We obtained the genotype data used for estimation of pairwise parental genetic similarity from the study by Sunde, et al. [32] where the relative performance of the two marker types (microsatellites and RADseq SNPs) to detect genetic structure was evaluated. The microsatellite data was retrieved from the Dryad Digital Repository [66], and RADseq SNPs from NCBI Sequence Read Archive (BioProject accession code PRJNA586770) [67]. In the study by Sunde, et al. [32], individuals were genotyped for ten microsatellite loci and 1580 SNPs. All ten microsatellites were found to be neutral, and the full RADseq dataset also reflected neutral evolution. In the present study we therefore used these datasets to represent partial neutral (microsatellites) and genome-wide neutral variation (the dataset referred to as ‘neutral SNPs’). Sunde, et al. [32] further searched for adaptive SNPs with multiple outlier analyses, including tests of locus-specific effects and gene-environment associations (GEAs). Because of the low number of populations included in their study, the tests of locus-specific effects suffered from low statistical power and were not able to detect any signals of selection despite clear indications from previous common garden and translocation studies that the populations are adaptively differentiated [36, 42–45]. In the GEA analysis with latent factor mixed model (LFMM), on the other hand, Sunde, et al. [32] detected loci associated with two environmental variables of importance for pike (salinity in the spawning ground during spawning and temperature at initiation of spawning) were identified. In the present study we therefore used the loci that they identified as outliers in the LFMM analyses to represent adaptive variation (the dataset referred to as ‘adaptive SNPs’). However, in the present study we chose to use a somewhat more liberal approach for classifying SNPs as outliers (adaptive) than used in the original study by Sunde, et al. [32] (in the present study we used a *P*-value cut-off of 0.01 instead of applying FDR correction for multiple testing) to not exclude potential outliers, and to increase the number of included outlier loci. This resulted in a total of 17 loci identified as putatively under selection (for details see Table S1 in Additional file 1). Based on the three datasets (microsatellites, neutral SNPs and adaptive SNPs), we then estimated pairwise parental similarity as the proportion of alleles shared between the two individuals in each family. This was calculated separately for each of the three datasets using R Studio v1.1.383 [68] with R v.3.2.2 [69].

### Statistical analysis

General or generalized linear mixed models (depending on the distribution of response variables) were used to test whether and how parental similarity varied among the three marker types (microsatellites, neutral SNPs, and adaptive SNPs), whether hatching success was associated with parental similarity, and to explore whether and how associations between hatching success and parental similarity were affected by admixture treatment (differed between purebred and admixed families). For this, we used the lme4 package v1.1–15 in RStudio with R. The different models were chosen based on the response distribution of the data: general linear mixed models for parental similarity (with a normal response distribution) and generalized linear mixed models with a logit-link function for hatching success (with a binomial response distribution).

For all tests, we treated marker type as a fixed categoric factor, parental similarity as a fixed continuous factor, and family (female/male pair) as a random factor. Statistical significance was assessed with Type III partitioning and an α–level of 0.05, and the Satterthwaite’s method was used to approximate degrees of freedom. For tests of interactions between factors, we excluded the interaction term and rerun the analysis in case of no significant interaction effect. For tests with significant terms, we further analyzed the data with Student’s t-test with non-pooled SD or Tukey’s test to determine the nature of the interactions and evaluate pairwise differences. *P*-values were adjusted using the FDR method [70] to account for multiple comparisons.

## Supplementary Information

**Additional file 1: Table S1**. Information on the 17 outlier loci that were used as adaptive dataset in the present study.

## Data Availability

The datasets supporting the conclusions of this article were obtained from previously pulished studies and are publicly available in Dryad Digital repository (Microsatellite genotype data: 10.5061/dryad.31zcrjdgv [66]; data on hatching success: file “offspring.xlsx”, 10.5061/dryad.dd64hf3 [46]), and the NCBI Sequence Read Archive (RADseq gentype data: BioProject PRJNA579326, https://www.ncbi.nlm.nih.gov/sra/PRJNA586770 [67]).

## Abbreviations

FDR: False discovery rate
LFMM: Latent factor mixed model
PCA: Principal component analysis
RADseq: Restriction-site associated DNA sequencing
SD: Standard deviation
SNP: Single-nucleotide polymorphism
