# Supplementary material for: How well do genetic markers inform about responses to intraspecific admixture? A comparative analysis of microsatellites and RADseq
Source: BMC Genom Data. 2021 Jun 28;22:22. doi: 10.1186/s12863-021-00974-3 (PMC8237422; doi:10.1186/s12863-021-00974-3)
Supplement: Supplementary file 1 — Additional file 1: Table S1. Information on the 17 outlier loci that were used as adaptive dataset in the present study. [file 12863_2021_974_MOESM1_ESM.docx]

# Supplementary materials for

How well do genetic markers inform about responses to intraspecific admixture? A comparative analysis of microsatellites and RADseq

*Yeşerin Yıldırım, Anders Forsman and Johanna Sunde**

***** Corresponding author. E-mail: johanna.sunde@lnu.se, Phone: +46-(0)480-446743

**Table S1** Information on the 17 outlier loci identified with the latent factor mixed model (LFMM) approach in the study by [1] that we used as adaptive dataset in the present study.

| **NCBI Genomic accession** | **Linkage group** | **Position** |
| --- | --- | --- |
| NC_025969.3 | LG02 | 619349 |
| NC_025977.3 | LG10 | 24790291 |
| NC_025977.3 | LG10 | 24790443 |
| NC_025971.3 | LG04 | 26328395 |
| NC_025974.3 | LG07 | 25209337 |
| NC_025974.3 | LG07 | 39563826 |
| NC_025977.3 | LG10 | 20011249 |
| NC_025977.3 | LG10 | 20011440 |
| NC_025977.3 | LG10 | 26802243 |
| NC_025979.3 | LG12 | 20186679 |
| NC_025980.3 | LG13 | 33064843 |
| NC_025981.3 | LG14 | 22726941 |
| NC_025982.3 | LG15 | 6181006 |
| NC_025982.3 | LG15 | 24890917 |
| NC_025986.3 | LG19 | 11458289 |
| NC_025990.3 | LG23 | 8065063 |
| NC_025990.3 | LG23 | 8656938 |

**Reference**

1. Sunde J, Yildirim Y, Tibblin P, Forsman A. Comparing the performance of microsatellites and RADseq in population genetic studies: analysis of data for pike (*Esox lucius*) and a synthesis of previous studies. Front Genet. 2020, doi: 10.3389/fgene.2020.00218.
